# Retraction: The MicroRNA-217 Functions as a Potential Tumor Suppressor in Gastric Cancer by Targeting GPC5

**DOI:** 10.1371/journal.pone.0283151

**Published:** 2023-03-29

**Authors:** The PLOS ONE Editors

The *PLOS ONE* Editors retract this article [1] because it was identified as one of a series of submissions for which we have concerns about peer review, authorship, and similarities across articles. Image similarities were noted between Fig 3E and Fig 5C in [1], and Fig 5D in [2]. These concerns call into question the validity and provenance of the reported results. We regret that the issues were not addressed prior to the article’s publication.

All authors either did not respond directly or could not be reached.

## References

[1] Wang H, Dong X, Gu X, Qin R, Jia H, Gao J (2015) The MicroRNA-217 Functions as a Potential Tumor Suppressor in Gastric Cancer by Targeting GPC5. PLoS ONE 10(6): e0125474. 10.1371/journal.pone.0125474 26098560 PMC4476558

[2] Li Z., Yu X., Wang Y., Shen J., Wu W. Ka Kei, Liang J., Feng F. By downregulating TIAM1 expression, microRNA-329 suppresses gastric cancer invasion and growth. Oncotarget. 2015; 6: 17559–17569. Retrieved from https://www.oncotarget.com/article/2755/text/ 25654811 10.18632/oncotarget.2755 PMC4627328

